# Structure and Magnetic Properties of Vanadium-Doped Heusler Ni-Mn-In Alloys

**DOI:** 10.3390/nano15191466

**Published:** 2025-09-24

**Authors:** Dmitry Kuznetsov, Elena Kuznetsova, Alexey Mashirov, Alexander Kamantsev, Denis Danilov, Georgy Shandryuk, Sergey Taskaev, Irek Musabirov, Ruslan Gaifullin, Maxim Kolkov, Victor Koledov, Pnina Ari-Gur

**Affiliations:** 1 Kotelnikov Institute of Radioengineering and Electronics of Russian Academy of Sciences, 125009 Moscow, Russia; kuznetsov.dmitry89@gmail.com (D.K.); a.v.mashirov@mail.ru (A.M.); kaman4@gmail.com (A.K.); 2 M.N. Miheev Institute of Metal Physics of Ural Branch of Russian Academy of Sciences, 620108 Ekaterinburg, Russia; monocrist@imp.uran.ru; 3 IRC Nanotechnology, Research Park, St. Petersburg State University, 199034 St. Petersburg, Russia; d.danilov@spbu.ru; 4 A.V. Topchiev Institute of Petrochemical Synthesis of Russian Academy of Sciences, 119991 Moscow, Russia; gosha@ips.ac.ru; 5 Faculty of Physics, Chelyabinsk State University, 454001 Chelyabinsk, Russia; tsv@csu.ru; 6 Institute for Metals Superplasticity Problems of Russian Academy of Sciences, 450001 Ufa, Russia; irekmusabirov@imsp.ru (I.M.); gaifullin_1998@bk.ru (R.G.); 7 Petersburg Nuclear Physics Institute Named by B.P. Konstantinov of National Research Centre «Kurchatov Institute», 188300 Gatchina, Russia; maxim_91@mail.ru; 8 Western Michigan University, Kalamazoo, MI 49008, USA; pnina.ari-gur@wmich.edu

**Keywords:** meta-magnetostructural transitions, martensitic transformation, Heusler alloys, microstructure

## Abstract

The crystal structure, texture, martensitic transformation, and magnetic properties of magnetic shape-memory Heusler alloys of Ni_51−x_Mn_33.4_In_15.6_V_x_ (x = 0; 0.1; 0.3; 0.5; 1) were investigated. Experimental studies of the magnetic properties and meta-magnetostructural transition (martensitic transition—MT) confirm the main sensitivity of the martensitic transition temperature to vanadium doping and to an applied magnetic field. This makes this family of shape-memory alloys promising for use in numerous applications, such as magnetocaloric cooling and MEMS technology. Diffuse electron scattering was analyzed, and the structures of the austenite and martensite were determined, including the use of TEM in situ experiments during heating and cooling for an alloy with a 0.3 at.% concentration of V. In the austenitic state, the alloys are characterized by a high-temperature-ordered phase of the L2_1_ type. The images show nanodomain structures in the form of tweed contrast and contrast from antiphase domains and antiphase boundaries. The alloy microstructure in the temperature range from the martensitic finish to 113 K consists of a six-layer modulated martensite, with 10 M and 14 M modulation observed in local zones. The morphology of the double structure of the modulated martensite structure inherits the morphology of the nanodomain structure in the parent phase. This suggests that it is possible to control the structure of the high-temperature austenite phase and the temperature of the martensitic transition by alloying and/or rapidly quenching from the high-temperature phase. In addition, attention is paid to maintaining fine interface structures. High-resolution transmission electron microscopy showed good coherence along the austenite–martensite boundary.

## 1. Introduction

Heusler alloys of the Ni-Mn-In system can exhibit unusual physical properties due to the characteristics of their atomic and electronic structure and magnetic behavior. These alloys demonstrate functional properties such as the shape memory effect (SME), the ferromagnetic shape memory effect (FSME), and the direct and inverse magnetocaloric effect (MCE) [1,2,3,4,5]. They owe their properties to the complex magnetic behavior with adjustable phase transition (PT) characteristics, achievable by changing their composition [6,7]. As with other Heusler alloys, their structure and PT affect their properties, which, as a result, determines their functionality. In addition to the chemical composition of the alloy, the chemical order in the crystal critically affects their potential. All these parameters provide further prospects for controlling the functional properties of these compounds and their practical application. Experimental efforts are mainly aimed at increasing the magnetization difference (∆M) between possible phases in Ni-Mn-based ferromagnetic shape-memory alloys by changing their stoichiometry, substituting atoms, and applying external pressure [8,9].

However, a number of unsolved problems remain for a thorough understanding of Ni-Mn-In alloys. One of them is the mechanisms of PTs A2–B2, B2–L2_1_, and austenite–martensite. For individual compositions, the specific characteristics of the transitions and their stages have not yet been established. The relevance of the study of Heusler Ni-Mn-In alloys is also determined by the need to establish the relationship between the properties and the fine structure that provides them. Some examples of theoretical and experimental studies of local atomic states of Heusler alloys were reported in Refs. [10,11,12,13].

Alloys undergoing martensitic transition (MT) are extremely sensitive to the degree of alloying with the elements that make up their composition. Alloying can lead to structural changes, segregation, decomposition, nanostructuring, and other effects that impact the characteristics of the alloys. It is known that changing the chemical composition and alloying in Heusler alloys changes the MT temperature (in the case of our system, this is also a meta-magnetostructural phenomenon; for simplicity, this is called MT in this paper) and the magnetostructural properties of the alloys, i.e., adding atoms of the fourth element can change the microstructure and magnetic response, the transition temperature, and the degree of hysteresis associated with the martensitic transition. Thus, one of the main tasks is to improve the magnetic properties of the alloys. For example, in Ref [14], it was shown that replacing Ni with Cu in the Ni_46−x_Cu_y_Mn_43_Sn_11_ alloy leads to a shift in the martensitic transition to a lower temperature; however, when replacing Sn with Cu in the Ni_16_Mn_12_Sn_4−x_Cu_x_ alloy, the transition shifts to a higher temperature [15] and a change in the response to an external magnetic field is observed.

In the development and use of many functional materials, it is necessary to characterize the structural details at several scale levels. In Ref [16], the effect of vanadium doping in the Ni_51−x_Mn_33.4_In_15.6_V_x_ system was shown; the authors observed a very low thermal hysteresis of 2.3 K in the Ni-Mn-In-based Heusler alloys through a strategy of introducing V. Together with a strong magnetostructural coupling, the reversibility of magnetic field-induced structural transitions is improved for a low field change of 0–1 T in the composition Ni_50.7_Mn_33.4_In_15.6_V_0.3_. Thus, a highly reversible MCE could be fulfilled. According to magnetic and caloric measurements, a considerable reversible Sm of 5.1 J kg^−1^ K^−1^ is obtained at 278 K for a field change of 0–1 T field variation. In addition, a large reversible Tad of −1.2 K is achieved for this field change. On the basis of a crystallographic analysis, the transformation stretch tensor is calculated, which indicates a good geometric compatibility between the two phases in Ni_50.7_Mn_33.4_In_15.6_V_0.3_. This compatibility is found to be responsible for the small hysteresis. Our work provides a feasible method for the practical application of Ni-Mn-X-based Heusler alloys as magnetocaloric materials. However, the structural characterization of the alloys was based solely on X-ray structural analysis. In this paper, we describe the structure based on methods that provide an understanding spanning the range from micrometer-phase segregation to nanometer structure. Transmission electron microscopy combined with electron diffraction and scanning electron microscopy with EBSD were chosen as the main methods for structural investigation. This method is the most informative for studying homo- and heterophase systems, their microstructure, relative orientation of phases, crystal structures, and defect distribution, including long-range and short-range order effects. This method was also useful for studying the Ni-Mn-In system.

The authors of the current work suggest that the formation of austenite after homogenization annealing, aimed at the uniform distribution of all chemical elements by concentration, occurs following a spinodal mechanism.

Spinodal decomposition is one of two possible mechanisms of phase separation in which a homogeneous thermodynamically unstable solution (solid or liquid) of two or more components separates into coexisting phases that differ in composition and, accordingly, in chemical and physical properties [17]. Spinodal decomposition is of great interest from a practical point of view. Metamaterials that are formed as a result of spinodal decomposition have a periodic fine-grained microstructure, which, in many cases, allows for a significant improvement in the physical properties of the material. The reason for this is that decomposition occurs uniformly throughout the volume of the material, by diffusion, without the formation of nuclei [18].

At present, systems with spinodal decomposition have been discovered in almost all classes of inorganic compounds, which makes it possible to make a metastable single-phase solid solution, consisting of two volume fractions, more thermodynamically stable and can be used as a basis for creating materials that are composites with unusual optical, magnetic, and electrical properties, as well as other functional characteristics [19]. The use of spinodal decomposition in the technology of inorganic materials to improve and enhance functional properties is in high demand and has important applications, but it requires deep fundamental and practical knowledge. The decomposition of solid solutions is associated with many phenomena, which must be considered in technical applications. In the process of the decomposition of solid solutions, various intermediate metastable states can arise, which are a single-phase solution with chemical heterogeneities in composition. Such states include phenomena called clusters, segregations, and Guinier–Preston zones [20]. The size, shape, and period of the distribution of new phase inclusions or concentration inhomogeneities are mostly determined by the mode of thermomechanical processing of the material. Thus, understanding the physics of decay processes can allow this phenomenon to be used as an effective method for obtaining ordered nanostructures with complex morphology in a virtually unlimited volume of materials.

The phenomenon of spinodal decomposition has been recorded in Heusler alloys, for example, in the Ni-Mn-In system [21,22,23,24], but there is very little information in the literature on the nature, kinetics, and mechanism of the process. Understanding the processes that lead to the formation of the austenite phase in Heusler alloys will allow us to fully reveal their functional and technological potential. According to the authors, spinodal decomposition is one of the key phenomena responsible for the structure and morphology of austenite in Heusler alloys, since the formation of the heterostructure in our case occurs through diffusion, and the structural components differ in the degree of ordering and, accordingly, chemical composition, without low- or high-angle boundaries (that is, the mechanism of nucleation and growth is excluded), determining the further course of phase transitions and related functional properties, such as SME, magnetic memory, MCE, ECE, etc. This work is devoted to a detailed structural analysis of the Heusler alloy as a promising functional alloy for micro- and nanosized mechanical manipulation devices.

## 2. Materials and Methods

Polycrystalline samples of nominal compositions (at. %) of Ni_51−x_Mn_33.4_In_15.6_V_x_ alloys (x = 0; 0.1; 0.3; 0.5; 1) were synthesized, using high-purity elements (>99.98%), using the arc melting method under an argon atmosphere on a chilled copper mold. The alloy was turned four times and remelted with currents of 100 A (to avoid the cracking of the ingot) for the first and second remelting and at 200 A for the third and fourth remelting; it remained in the molten state for 2 min to improve homogeneity. An EDX analysis of the ingots after smelting was carried out at 10 points along the ingot. The EDX analysis results are summarized in Table 1. In total, two batches of samples from five compositions were smelted.

For further homogenization, the ingots were annealed in a vacuum at a temperature of 1173 K for 48 h, followed by quenching in cold water with an average cooling rate, which was calculated as (1173 K − 300 K)/437 min = 1.997 K/min.

The resulting structure was studied in the temperature range of 113 to 323 K by means of a Carl Zeiss Libra 200FE (Oberkochen, Germany) transmission electron microscope (TEM) (using an accelerating voltage of 200 kV) with an energy OMEGA filter, an Oxford Instruments X-Max 80 energy-dispersive X-ray detector (GB), and a Gatan Model 636 two-axis cryoanalytical holder (GB) with a Model 900 SmartSet cold stage controller (GB). The detector was a high-angle dark-field scanning transmission electron microscope (HAADF-STEM).

Electron backscatter diffraction (EBSD) (GB) studies were performed using a Carl Zeiss Merlin (Germany scanning electron microscope equipped with an Oxford Instruments CHANNEL5 (GB) electron backscatter diffraction recording system. The accelerating voltage was 30 kV and the beam current was 5 nA. To construct EBSD maps, a sequential study of a selected sample area was carried out with a step size of 4 μm and 0.2 μm). The processing and analysis of EBSD maps were performed using Tango 9.3.4 software.

The characteristic temperatures (for the bulk sample) of the beginning and end of the forward and reverse thermoelastic MTs and the Curie temperature were determined via differential scanning calorimetry (DSC) on a METTLER TOLEDO DSC3+ (Greifensee, Switzerland) setup in the temperature range from 100 to 600 K at a speed of 10 K/min.

The magnetic properties were studied by standard methods using a Quantum Design vibration magnetometer (San Diego, CA, USA) in the zero-field cooling—field heating—field cooling (ZFC–FH–FC) mode, with a temperature range of 50 to 400 K. The measurement accuracy of the magnetic moment of the Vibrating Sample Magnetometer of this measuring system is ±0.5%.

## 3. Results and Discussion

### 3.1. Thermal Measurements

**Figure 1:**
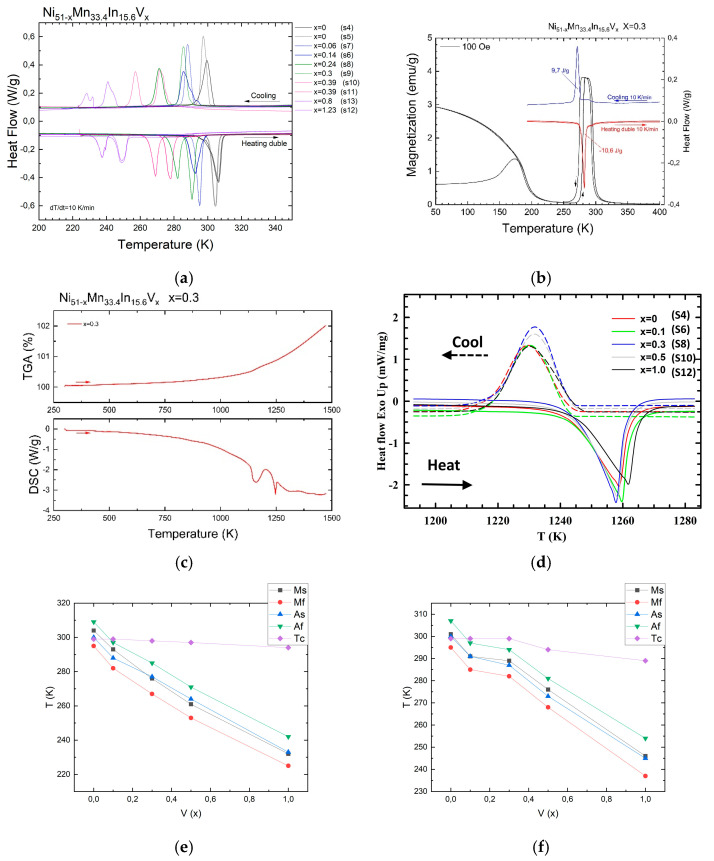
(**a**) DSC thermograms of Heusler Ni_51−x_Mn_33.4_In_15.6_V_x_ alloys (x = 0; 0.1; 0.3; 0.5; 1); (**b**) comparison of DSC and magnetization data for the Ni_51−x_Mn_33.4_In_15.6_V_x_ alloy (x = 0.3); (**c**) medium- and high-temperature DSK and TGA to determine the order–disorder PT temperature (B2–L2_1_) of the Ni_51−x_Mn_33.4_In_15.6_V_x_ alloy (x = 0.3); (**d**) high-temperature DSC curves that reveal the melting and crystallization temperatures; (**e**) dependence of the characteristic temperatures of the martensitic transformation and the Curie temperature on the vanadium doping level for samples S4, 6, 8, 10, and 12; (**f**) dependence of the characteristic temperatures of the martensitic transformation and the Curie temperature on the vanadium doping level for samples S5, 7, 9, 11, and 13.

From the DSC data (Figure 1), the presence of the following PTs is observed: a low-temperature MT; the hysteresis is calculated using the formula ΔT_hys_ = (A_s_ + A_f_ − M_s_ − M_f_)/2) and a Curie temperature Tc = 298 K (Figure 1a), with an order–disorder PT (B2–L2_1_) at a temperature of 1150 K (Figure 1c) and a melting temperature of 1260 K (Figure 1c). As can be seen from Figure 1e,f, an increase in the vanadium doping level leads to a decrease in both direct and reverse MT temperatures. The Curie temperature is less dependent on the vanadium doping level, but a linear and monotonic decrease is also noticeable.

**Figure 2:**
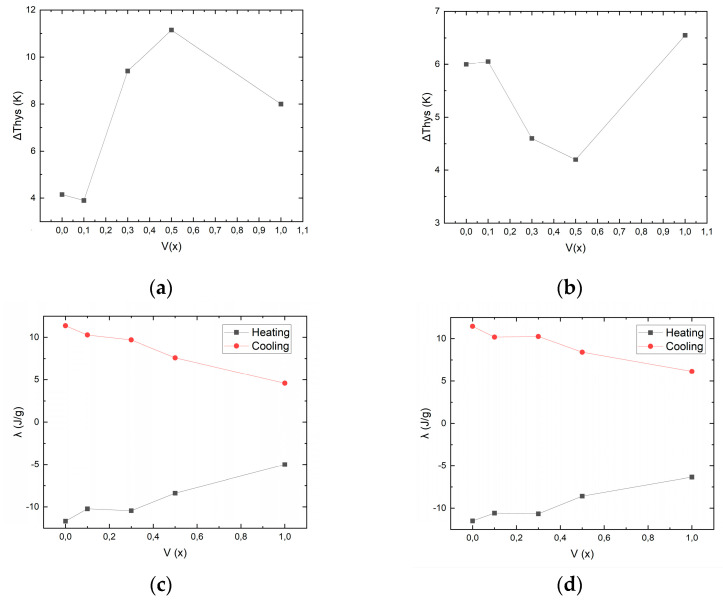
Dependences of the hysteresis width and latent heat of MT on the vanadium doping level: (**a**,**c**) samples S4, 6, 8, 10, and 12; (**b**,**d**) samples S5, 7, 9, 11, and 13.

The dependence of the hysteresis width on the vanadium doping level is shown in Figure 2a,b, while that of the latent heat of MT is shown in Figure 2c,d.

For both series of samples, it is evident that the latent heat of MT decreases linearly and monotonically with increasing vanadium doping levels. On the other hand, the nature of the dependence of the hysteresis width on the vanadium doping level is inconsistent and such a spread of values is apparently due to the heterogeneity of the alloy structure, namely, vanadium that has not been completely dissolved; this will be described in detail below.

### 3.2. Magnetic Measurements

**Figure 3:**
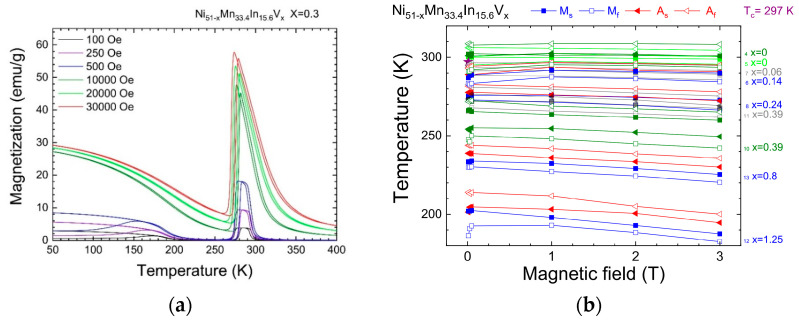
(**a**) Field dependence of magnetization of alloy S8 (x = 0.3); (**b**) shift in the direct and reverse MT temperatures. Fields and compositions are indicated in the figures.

An example of the field dependence of the magnetization at different temperatures for an alloy with a V content of x = 0.3 at. % is shown in Figure 3a (the remaining graphs are presented in Appendix A). The presence of MT and the high sensitivity of the characteristic temperatures of the meta-magnetostructural PT to magnetic field are shown.

M_s_ and M_f_—martensite start and finish temperatures;

A_s_ and A_f_—austenite start and finish temperatures.

The shift in the characteristic temperature of the MT due to an applied magnetic field is k_Ms_ = +1 K/T from 0 to 1 T and k_Ms_ = −1.6 K/T from 1 to 3 T (Figure 3b). This provides the possibility of using this alloy in magnetically and thermally controlled actuators of microelectromechanical systems.

### 3.3. Structural Analysis

#### 3.3.1. X-Ray Diffraction Analysis

**Figure 4:**
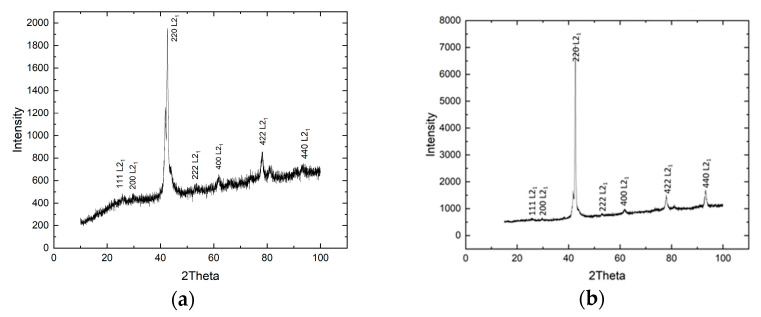
X-ray diffraction patterns of alloys with vanadium doping levels of x = 0.3 at. % (**a**) and x = 0.5 at % (**b**).

Examples of X-ray diffraction patterns for alloys containing 0.3 and 0.5 at. % vanadium are shown in Figure 4 The measurements were taken at room temperature (approximately 300 K), which is above the A_f_ temperature, i.e., in the austenitic state. The diffraction peaks were indexed, assuming a cubic L2_1_ type. The Match!3 program did not detect the presence of any secondary phases, including the presence of pure vanadium and its compounds. This program does not provide adequate data on the lattice parameter, since it does not collect a sufficient number of peaks for determination.

#### 3.3.2. Electron Microscopy

**Figure 5:**
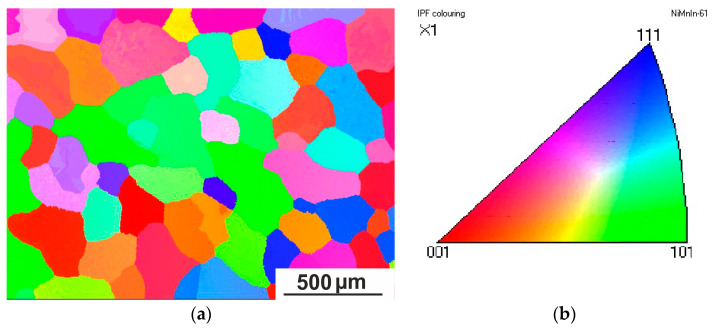
Orientation EBSD map of austenite of Ni_50.0_Mn_33.4_In_15.6_V_1.0_ alloy with a scanning step of 4 μm (**a**) and the inverse pole figure (**b**). Observations at 300 K.

A grain orientation map of the austenite of the quenched Ni_50.0_Mn_33.4_In_15.6_V_1.0_ alloy was obtained by EBSD analysis at 300 K (Figure 5). Each grain has its own orientation. The grain size reached hundreds of microns (200–500 μm). It is worth noting that in addition to recrystallized grains, abnormally large grains of 3 mm or more in size were also observed, indicating the occurrence of collective recrystallization.

**Figure 6:**
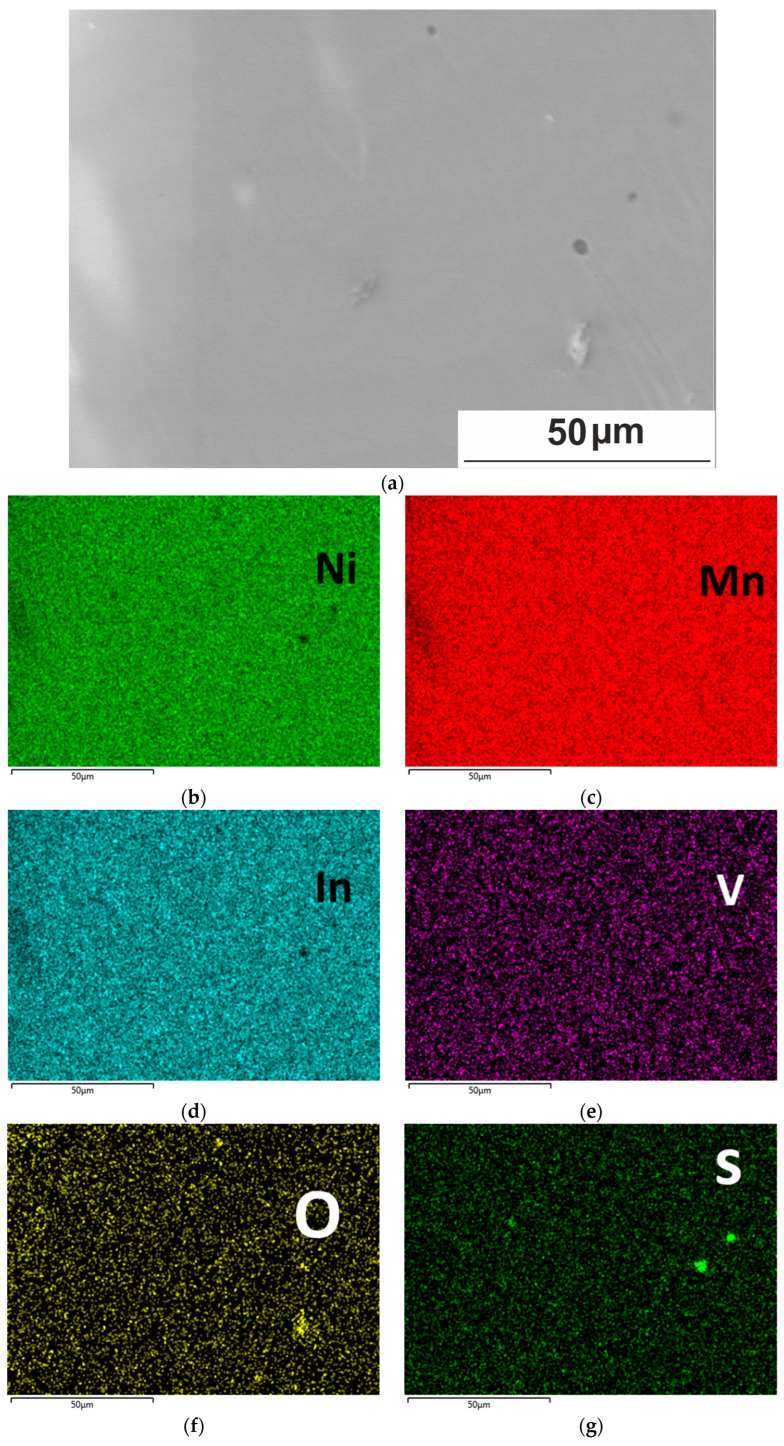
Element distribution maps (EDX) at 300 K of the Ni_50.7_Mn_33.4_In_15.6_V_0.3_ alloy: SEM image of the structure (**a**), maps of the distribution of chemical elements, element labels are indicated in the figures (**b**–**g**).

In common melting methods such as arc melting, non-metallic inclusion particles may form during solidification due to C and O impurities introduced into the melt either by the initial pure metals or during the melting process itself. Mapping the alloy surface in the characteristic X-ray spectra of chemical elements Ni, Mn, In, V, S, and O, it was shown that the main components of the alloy—nickel, manganese, and indium—were uniformly distributed, while oxygen and sulfur were concentrated in inclusions (Figure 6). As for vanadium, the effects of clustering or segregation at low levels of the alloying are difficult to distinguish. The intensity enhancements in the S map coincide with the enhancements of the Mn signal and depletions of Ni and In. The brightest local area on the O map of Figure 6e, located in the lower right corner, may correspond to the oxides of Ni, Mn, and In. Thus, the alloy is characterized by the presence of oxides and sulfides that apparently formed during ingot melting. From the microanalysis, it can be observed that the chemical composition in the excess phase region differs by an excess of Mn and a lower content of Ni and In relative to the nominal value. Sulfur and oxygen were not added intentionally to the material. However, both elements are common impurities in commercial precursors and can enter from the atmosphere during processing. Vanadium can form carbides at temperatures of 1073–1273 K during heat treatment, as well as during heat treatment at lower temperatures [25,26] and even during the melt forming process and can also react with nitrogen at temperatures above 1073 K.

**Figure 7:**
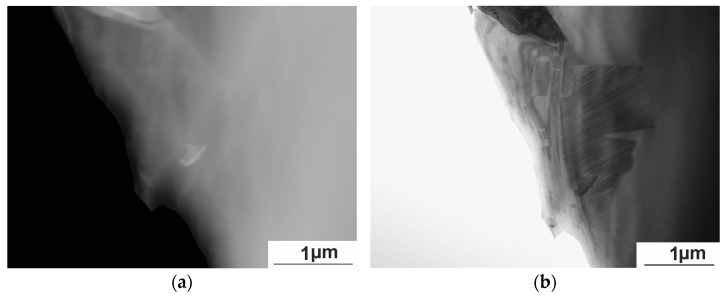
High-angle annular dark-field imaging (HAADF) of the structure of the Ni_50.7_Mn_33.4_In_15.6_V_0.3_ alloy at 300 K (**a**) and at 113 K (**b**).

As can be seen from Figure 7, the presence of inclusions has virtually no effect on the mechanism of martensite plate formation and the inclusions are not crack nucleation centers. Dispersed carbides, sulfides, and oxides can hinder the nucleation and growth of martensite crystals with their elastic stress fields, while the precipitation of large, sparsely located particles, does not inhibit martensite formation [25,26].

**Figure 8:**
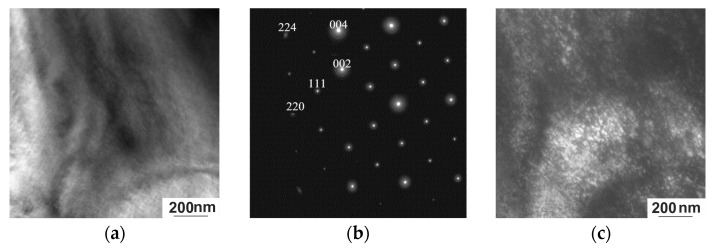
Structure of the Ni_50.7_Mn_33.4_In_15.6_V_0.3_ alloy at 300 K. (**a**) Bright-field image; (**b**) corresponding electron diffraction pattern, zone axis [110]; (**c**) dark-field image obtained in the [111] L2_1_ superstructure reflection.

In situ TEM observations were carried out in the range from 300 K to 100 K. Electron microscopy examination combined with Bragg reflections showed that the quenched Ni_50.7_Mn_33.4_In_15.6_V_0.3_ alloy exhibits a complex mixture of intense diffuse effects at room temperature. This is a long-wave diffuse scattering corresponding to transversely polarized waves of atomic displacements, which is accompanied by a tweed contrast in the images (Figure 8a). In the diffraction patterns, not only diffuse effects are observed but also superstructure reflections (Figure 8b). At the same time, ultra-disperse regions up to 20 nm in size are visible in the dark-field images obtained in superstructure reflections of the [111] type (Figure 8c).

**Figure 9:**
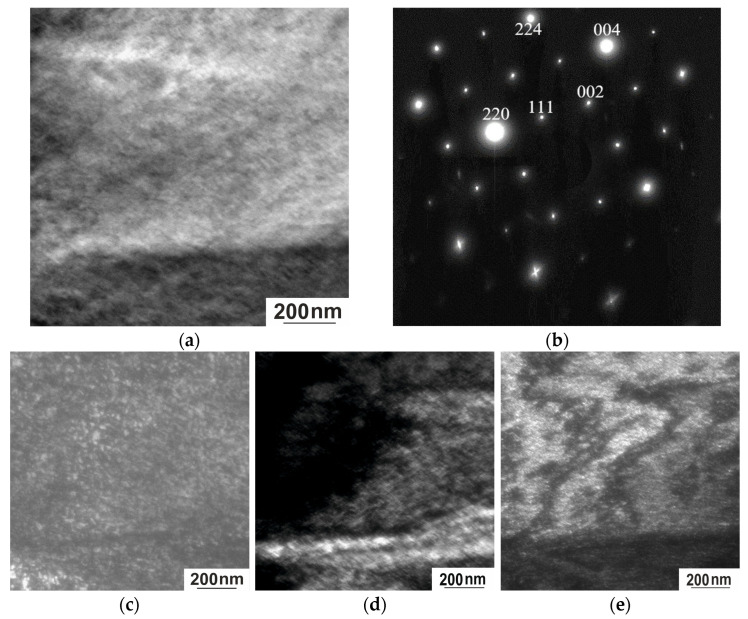
Structure of the Ni_50.7_Mn_33.4_In_15.6_V_0.3_ alloy at 300 K. (**a**) Bright-field image; (**b**) corresponding electron diffraction pattern, zone axis [110]; (**c**) dark-field image obtained in the superstructural reflection [111]_L21_; (**d**) dark-field image obtained in the reflection [004]_L21_; (**e**) dark-field image obtained in the reflection [220]_L21_.

The entire experimentally observed set of diffraction patterns (Figure 8 and Figure 9) is associated with a high-temperature ordered phase of the L2_1_ type, developed during quenching from high temperatures [21]. Characteristic superstructural reflections of the [111] type are also observed. The main diffraction spots are well indexed by a cubic lattice with a lattice parameter a = 5.88 Å. The ordered nature of the L2_1_ phase and the diffusion mechanism of its formation are confirmed by the observation of antiphase domains and antiphase boundaries in superstructural reflections in electron microscopic images [14]. In the dark-field images obtained in superstructural reflections [111] (Figure 8c and Figure 9c), the shape of the ordered domains in the foil plane is close to square, with a size of ~20 nm. The nanometer size of the domains results in the high density of antiphase boundaries. The width of the contrast of the images of antiphase boundaries (APBs) is almost commensurate with the size of the antiphase domains (APDs), which demonstrates the effect of the blurring of the APBs, which increases with deviation from stoichiometry [27]. APDs with a size of ~20 nm exhibit thermal stability; after an additional low-temperature annealing at 323 K for 0.5 h (to relieve internal stresses formed during quenching), the size of the domains did not increase.

In the electron diffraction patterns of austenite, near the main L2_1_ reflections, diffuse scattering effects are observed in the form of two radial diffuse strands intersecting at an angle of ~115° along the [224]* direction. The tweed contrast bands are in accordance with the diffuse scattering bands, whereby each system of contrast bands is orthogonal to one of the diffuse scattering directions. The diffuse bands in the diffraction patterns of martensite are explained by the coexistence of several modulated structures (6 M, 10 M, and 14 M). Since the foil and the studied areas have a wedge-shaped form, thickness-related effects will inevitably arise, but they do not affect the interpretation of the data.

The contrast from the APD occurs in both superstructural and fundamental reflections, but its nature differs significantly (Figure 9c,d). This suggests that the nature of the contrast is due to ordering, which, in turn, causes a number of indirect effects. The most intense diffuse scattering in the form of diffuse strands along <112>* is observed near fundamental reflections of the 004, 220, and 224 types (Figure 9c). Therefore, in the dark-field image obtained in the fundamental reflection 004, both systems of tweed contrast with the long-wave component characteristic of this state are observed (Figure 9d). As noted previously, the regular nature of diffuse scattering and tweed contrast is due to the presence of localized cooperative, mainly shear, displacements of atoms in the crystals [28]. These phenomena indicate that the alloy under study is in a pre-transition state. Tweed contrast, considered as a pre-transition effect, has been observed by TEM in many materials, including shape-memory alloys [23], at the tetra-ortho transition in high-temperature cuprates of the YBa_2_Cu_3_O_7−δ_ type [29,30], and alloys undergoing phase separation.

**Figure 10:**
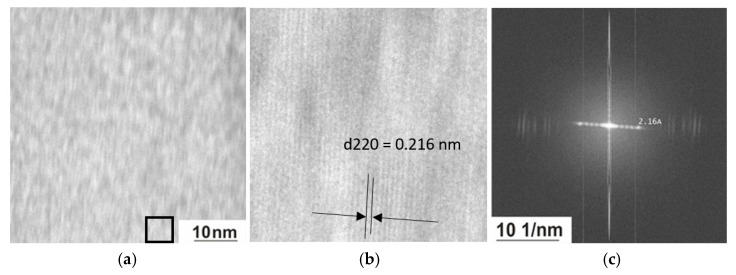
Domain structure in the Ni_50.7_Mn_33.4_In_15.6_V_0.3_ alloy (**a**), the image of domains in direct resolution from the area whitened by the black square (**b**), and the corresponding Fourier transformation (**c**) obtained at 300 K.

Figure 10 shows images obtained using TEM direct resolution mode and the corresponding Fourier transformation. One of the challenges of high-resolution transmission electron microscopy is that image formation depends on phase contrast. As is well known, in phase-contrast imaging, contrast is not intuitively interpretable because the image is affected by aberrations of the microscope objectives. The largest contributors to uncorrected images are usually defocus, spherical aberration, and objective astigmatism. In spite of the fact that the studied Heusler alloy is highly magnetic and has a complex structure and dynamic tweed contrast, we managed to obtain the direct resolution of atomic planes and determine the interplanar spacing. In the region under consideration, one more large-scale level of structural organization is observed—a nanodispersed domain structure (Figure 10a) and a crystalline phase with an interplanar spacing of 0.216 nm, which corresponds to d_220_ of the ordered L2_1_ phase. The striped contrast observed in Figure 10b is formed by projections of the {220} planes, where d_220_ = 0.216 nm. The key factor is the scale and periodic nature of the distribution of the fine domain structure. Potentially, this micrograph may correspond to both the domain structure arising during ordering and the early stage of decay. The characteristic scale of the domain structure is ~ 4 × 4 nm.

**Figure 11:**
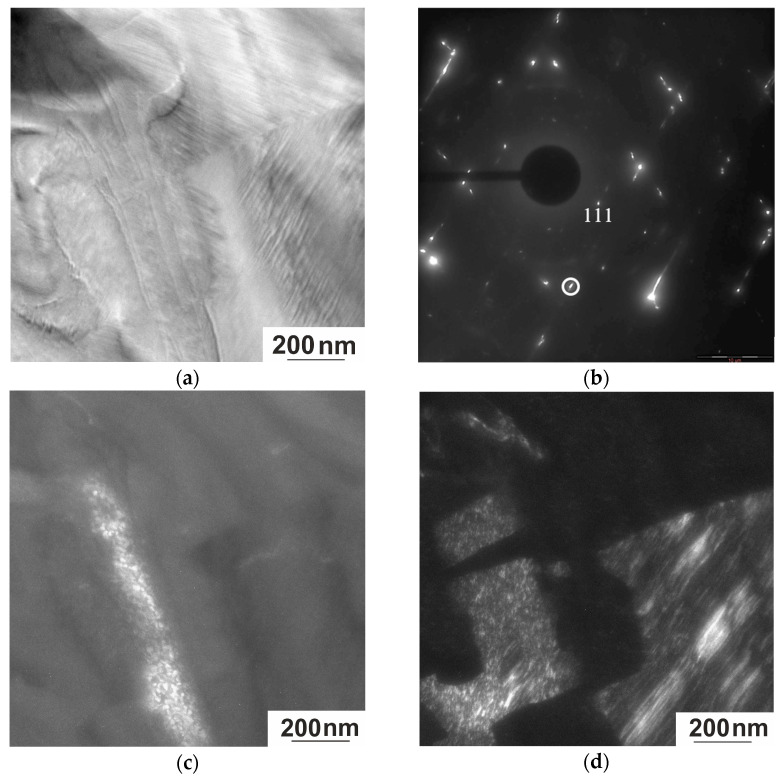
Structure of the Ni_50.7_Mn_33.4_In_15.6_V_0.3_ alloy at 113 K. (**a**) Bright-field image; (**b**) corresponding electron diffraction pattern, zone axis [110]_L21_; (**c**) dark-field image in reflection 111_L21_; (**d**) dark-field image in the selected (outlined in white) reflection.

Bright-field and dark-field images and the corresponding microelectron diffraction patterns of the quenched Ni_50.7_Mn_33.4_In_15.6_V_0.3_ alloy in the martensitic state are shown in Figure 11. The microstructure morphology is represented by packets of plate-like finely twinned martensite crystals. Extra reflections located at a distance of 1/6 between the main reflections indicate a multilayered lattice of the 6 M type (Figure 11b). Satellites are also observed at positions 1/5 and 1/7 between the reflections, which suggests the existence of other modulated structures (10 M and 14 M). Such irregularity explains the elongation of the satellites and diffuse bands in the diffraction patterns.

The diffraction pattern in Figure 11b is obtained from a region with two sets of micro-twins rotated relative to each other by approximately 80°, indicating an orientational relationship between the two martensite variants (right-hand side of Figure 11a). At least up to a temperature of 113 K, this alloy is in a two-phase state. From the interpretation of the electron diffraction patterns and the analysis of the dark-field images, it follows that the light region in Figure 11c (the dark-field image was obtained in the superstructural reflection 111_L21_) is residual austenite with L2_1_ order, and the banded contrast (lower right-hand side of Figure 11d) belongs to the 6 M phase.

**Figure 12:**
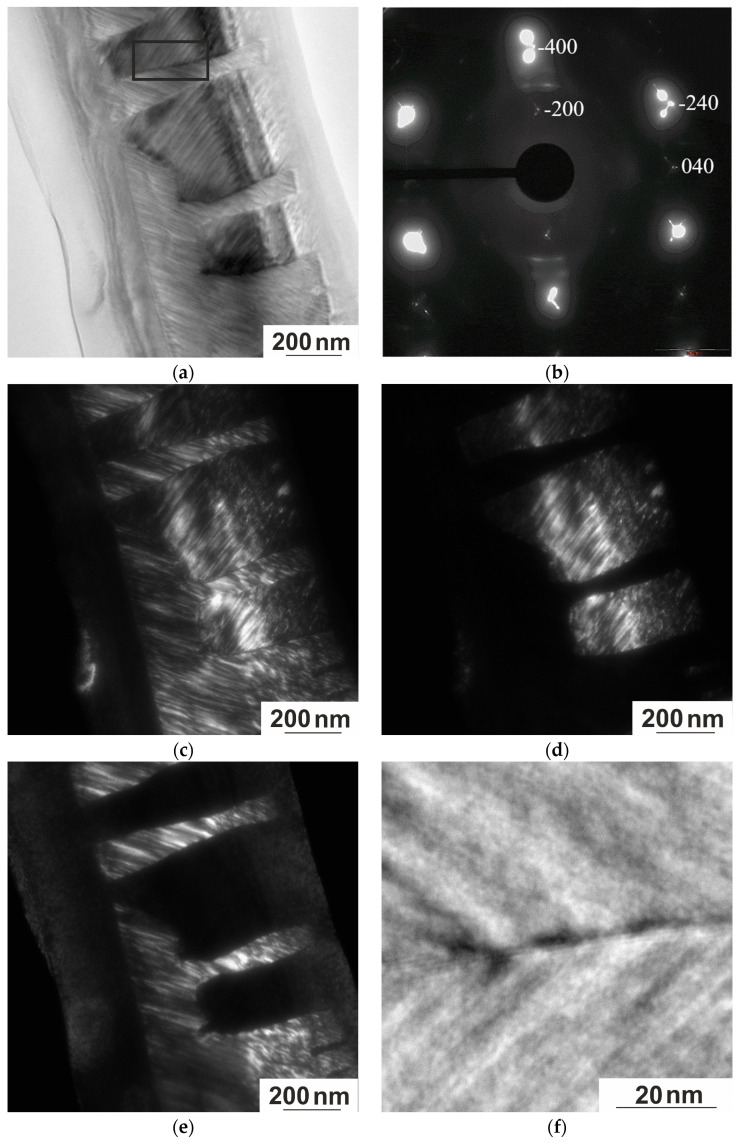
Structure of the Ni_50.7_Mn_33.4_In_15.6_V_0.3_ alloy at 113 K. (**a**) Bright-field image; (**b**) corresponding electron diffraction pattern, zone axis [001]_L21_; (**c**) dark-field image in the −400_L21_ reflection together with satellites; (**d**) dark-field image in the upper satellite of the −400_L21_ reflection; (**e**) dark-field image in the lower satellite of the −400_L21_ reflection; (**f**) enlarged bright-field image of the contact boundary region of the two types of martensite (marked by a black square in Figure 12a).

To consider the microstructural features associated with the formation of martensite variants of different zone axes orientations, a section with the <001> direction normal to the foil surface was used (Figure 12). Light-and dark-field micrographs are shown in Figure 12a,c,d, while the corresponding microdiffraction pattern is shown in Figure 12b. Analysis of the micrographs and microdiffraction patterns allowed us to determine that the martensite of the alloy under study consists of packets of pairwise twinned parallel plates with flat habit boundaries close to {110}_L21_, as well as thin secondary twins inside them with mutually orthogonal orientation in adjacent plates. The presence of packets of twins of different scales in the martensitic structure is a consequence of the mechanism of accommodation twinning and the formation of long-period shear martensite phases. This approach is described by the adaptive concept proposed in Ref [31] and developed in Ref [32,33]. It is assumed that the lattice mismatch formed on the habit plane can be compensated by the formation of a martensite nanotwin.

Although the boundaries between the martensitic plates appear thin and straight, HRTEM images reveal that these boundaries are, in fact, more complex. A thin transition layer of 4–10 nm thick banded structure with a spacing of ~2 nm between the bands is observed in the interface zone at the boundary between martensitic plates. An enlarged image of such a region is shown in Figure 12f. The boundary is mainly straight and contains up to five atomic layers, forming steps at local locations. The microtwin of one plate stops at the microtwin of the other plate, forming a step-shaped interface. The twin microstructure formed after the martensitic transformation is such that the size and shape of the martensite approach the size and shape of the domain structure of the preceding austenite; the size of the martensite twins can be used to judge the size of the APDs and vice versa.

It is plausible to assume the existence of a thin nanoscale layer of austenite L2_1_ at the boundaries of martensite blocks, which is confirmed by the preservation of superstructural reflections <111> in the diffraction patterns of martensite. In addition, the L2_1_ reflections overlap the reflections of martensite and can be interpreted as satellites caused by twinning. Thus, the diffraction pattern in Figure 12b can be attributed to the probable coexistence of two phases—L2_1_ and modulated martensite. Thin layers of the L2_1_ phase between martensite plates can be associated with the observed nanodomain structure (Figure 10), corresponding to the early stage of decomposition. The interaction of local areas of stratification by concentration can lead to the formation of thin layers of this phase along the boundaries of martensite plates.

**Figure 13:**
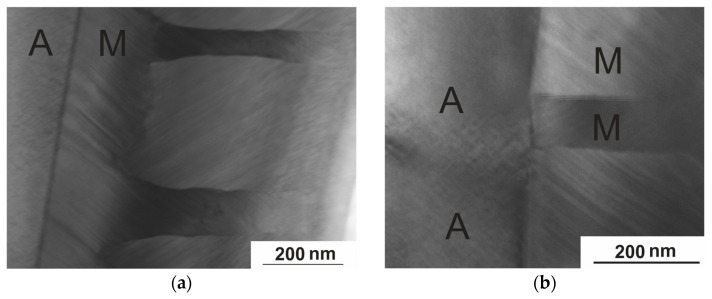
HAADF image of the A–M boundary of the Ni_50.7_Mn_33.4_In_15.6_V_0.3_ alloy at 113 K (**a**); direct resolution of the M–M boundary (**b**).

In addition to the comprehensive microstructural study, due to the presence of a critical value of the sample thickness at which the transition is completely suppressed [24], it was possible to study the interphase boundary between austenite and martensite (A–M). Varying the thickness of the TEM foil makes it possible to simultaneously observe both the martensitic phases known in the Ni–Mn–In system and the austenite (Figure 13a). In the austenitic phase detected near the interphase boundary between austenite and martensite, a premartensitic state is observed with a characteristic tweed microstructure and deformation fields formed as a result of local lattice deformations. In the diffraction patterns of the austenite, the intensity of the diffuse bands along <112>* that correspond to the tweed contrast gradually increases, while in the diffraction patterns of martensite, satellites appear on these bands at a distance of 1/6 to the main reflections. Figure 13b clearly shows that the microtwin structure of the martensite matches the domain size of the tweed structure of the austenite, and good coherence is observed across the A–M interface. In this paper, the coherence at the austenite–martensite interface is clearly demonstrated using TEM. The authors of Ref [16] provide data on the improvement of functional properties but do not consider the fine structure responsible for these effects. Our structural studies confirm the data in Ref [16]. This means that it becomes possible to reliably tune the magnetocaloric effect and the shape-memory effect (both effects are inextricably linked to the martensitic transition), which can be adjusted continuously by choosing a suitable aging parameter (heat treatment to form the required microstructure with specified parameters of temperature, holding time, and cooling rate). Similar effects were found in the study of MT in NiMnGa alloys [34], which determined that the modulated martensite structures of 10 M and 14 M were present only near the austenite–martensite interface, while unmodulated martensite was found to be primarily far from the interface. Figure 13b shows an enlarged image of the martensite platelet (M–M) boundary as a banded contrast of approximately five atomic layers.

**Figure 14:**
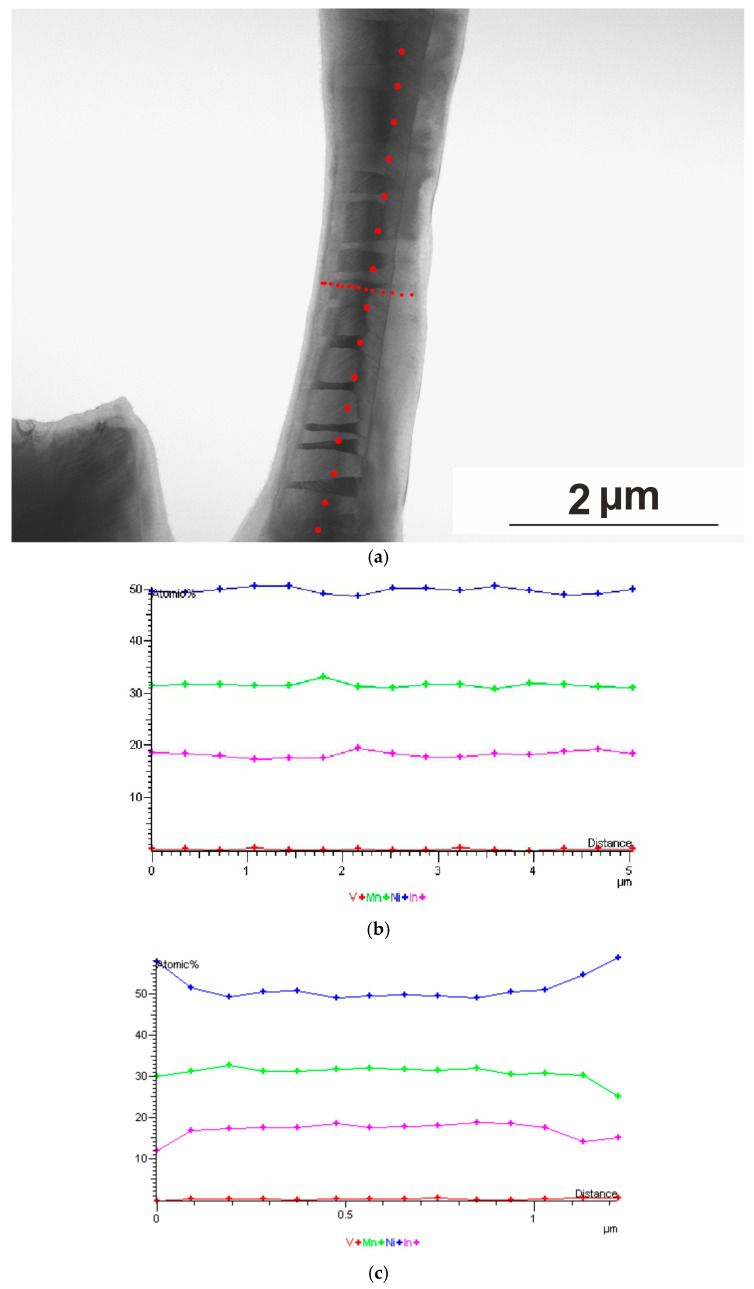
EDX spectra of the Ni_50.7_Mn_33.4_In_15.6_V_0.3_ alloy. (**a**) General view of the studied area at 113 K; red dots indicate the places where EDX spectra were obtained. (**b**) Distribution of elements along the long side of the studied area obtained at 300 K. (**c**) Distribution of elements obtained at 300 K.

Figure 14 shows the EDX (in line spectrum mode, taken at points along a given line at equal distances obtained at 300 K) spectra of a plate (3000 nm long, 1000 nm wide, and 50–100 nm thick), the structure of which is shown in Figure 12 and Figure 13. The EDX spectra indicate that all the original elements Ni, Mn, In, and V are present.

As mentioned earlier, the thermal response of the materials studied in this work depends strongly on the detailed microstructural features, which, in turn, can be a consequence of size effects. There are two potential sizes that can affect the properties of shape memory alloys—grain size and sample size. The effect of grain size is quite well understood, especially when the grain size is much finer than the sample size, as it has been shown that martensite formation is suppressed at smaller relative grain sizes due to their three-dimensional confinement. The commensurability of grain and sample sizes suggests that the effect of sample size on MT should be considered. In Ref. [16], the features of the metamagnetic martensitic transformation of the Ni_46_Mn_41_In_13_ alloy in a thin wedge-shaped plate were studied and it was found that the martensitic transformation is completely suppressed at a plate thickness less than 50 nm. In Ref. [35], the crystal structure of conical Ti_2_NiCu plates and the temperature Tc at which the MT occurs were investigated; it was found that the critical thickness value at which the transition is completely suppressed is 20 nm. There are very few studies on such size effects and those that exist are insufficient to draw general conclusions to determine the limiting values of the device size based on such alloys. Figure 10, Figure 11 and Figure 12 examine the microstructural features of the martensitic transformation in an object that is a thin plate (3000 nm long, 1000 nm wide, and 50–100 nm thick).

## 4. Conclusions

In this paper, the structure, martensitic transformation, and magnetic properties of Ni_51−x_Mn_33.4_In_15.6_V_x_ (x = 0; 0.1; 0.3; 0.5; 1) shape-memory alloys alloyed with vanadium are investigated. The results of the study allow us to draw the following conclusions:

1. The microstructure of the austenite of the Heusler alloy studied is formed by the spinodal decomposition mechanism, and not through nucleation and growth, since, in essence, the system is structurally divided into regions that differ primarily in the degree of ordering and, accordingly, chemical composition without low- or high-angle boundaries—an antiphase domain structure.
2. There is a microstructural connection between austenite and martensite. The type of modulated twin structure of martensite is determined by the high density of AFD and AFB in the initial austenite phase. The microtwin structure of martensite corresponds to the size of the domains of the tweed structure of austenite, and a defect-free boundary with good coherence of the A–M phases is observed.
3. The alloy microstructure consists predominantly of six-layer modulated martensite up to 113 K. Modulated structures of 10 M and 14 M martensite are also observed. An HRTEM study revealed a thin layer of 4–10 nm thick with a banded contrast of 2 to 5 atomic layers at the boundary of martensite packets.
4. The formation of multiple martensite variants undoubtedly affects the conditions for the formation of the MT. The shift in the temperature of the onset of martensitic transformation k_Ms_ above 1 T is −0.5 K/T, −1.6 K/T, −3.5 K/T for alloys with a nominal vanadium doping level of x = 0 (S4), 0.3 (S8), and 1 (S12) at.%, respectively, and the temperature hystereses (ΔT_hys_) are 5.6 K, 5.0 K, and 9.1 K, with an increase in width of about 0 K/T, 0.33 K/T, and 0.45 K/T.

## Figures and Tables

**Table 1:** EDX analysis of Ni_51−x_Mn_33.4_In_15.6_V_x_ alloys (x = 0; 0.1; 0.3; 0.5; 1).

№	Nominal Composition (at.%)	EDX Average Value, at. %. (Ni; Mn; In; V)	EDX Maximum Value, at. % (Ni; Mn; In; V)	EDX Minimum Value, at. % (Ni; Mn; In; V)	EDX Standard Deviation, at. % (Ni; Mn; In; V)
**S4**	Ni_51−x_Mn_33.4_In_15.6_V_x_ (x = 0)	50.38; 34.02; 15.60; 0	50.89; 35.14; 15.83; 0	49.51; 33.49; 15.35; 0	0.59; 0.50; 0.17; 0
**S5**	Ni_51−x_Mn_33.4_In_15.6_V_x_ (x = 0)	50.36; 34.04; 15.59; 0	50.75; 34.36; 15.67; 0	50.07; 33.64; 15.52; 0	0.28; 0.25; 0.06; 0
**S6**	Ni_51−x_Mn_33.4_In_15.6_V_x_ (x = 0.1)	50.19; 34.04; 15.63; 0.14;	50.37; 34.24; 15.94; 0.20;	49.88; 33.87; 15.40; 0.10;	0.04; 0.16; 0.17; 0.19
**S7**	Ni_51−x_Mn_33.4_In_15.6_V_x_ (x = 0.1)	50.34; 33.92; 15.69;0.06;	50.51; 34.26; 15.93; 0.15;	50.11; 33.69; 15.37; 0.00;	0.06; 0.24; 0.16; 0.22
**S8**	Ni_51−x_Mn_33.4_In_15.6_V_x_ (x = 0.3)	50.24; 33.87; 15.65; 0.24;	50.47; 34.06; 15.82; 0.31;	50.00; 33.65; 15.55; 0.18;	0.05; 0.15; 0.18; 0.11
**S9**	Ni_51−x_Mn_33.4_In_15.6_V_x_ (x = 0.3)	50.31; 33.79; 15.61; 0.30;	50.62; 34.09; 15.94; 0.48;	49.99; 33.54; 15.29; 0.22;	0.09; 0.21; 0.26; 0.23
**S10**	Ni_51−x_Mn_33.4_In_15.6_V_x_ (x = 0.5)	49.85; 34.03; 15.73; 0.39;	50.02; 34.14; 15.88; 0.50;	0.33; 33.88; 49.74; 15.57	0.07; 0.09; 0.12; 0.10
**S11**	Ni_51−x_Mn_33.4_In_15.6_V_x_ (x = 0.5)	49.95; 33.94; 15.72; 0.39;	50.24; 34.05; 15.86; 0.50;	49.73; 33.81; 15.57; 0.31;	0.08; 0.11; 0.24; 0.11
**S12**	Ni_51−x_Mn_33.4_In_15.6_V_x_ (x = 1)	49.80; 33.54; 15.43; 1.23;	50.12; 33.66; 15.71; 2.15;	49.28; 33.29; 15.08; 0.84;	0.49; 0.14; 0.29; 0.24
**S13**	Ni_51−x_Mn_33.4_In_15.6_V_x_ (x = 1)	49.93; 33.59; 15.68; 0.80;	50.14; 33.73; 15.94; 0.89;	49.80; 33.47; 15.55; 0.74;	0.06; 0.11; 0.12; 0.15

**Figure A1:**
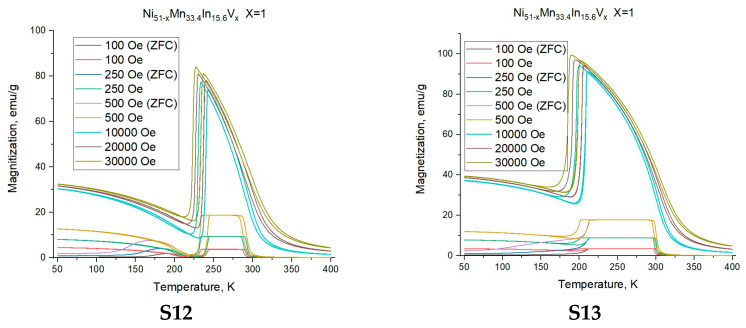

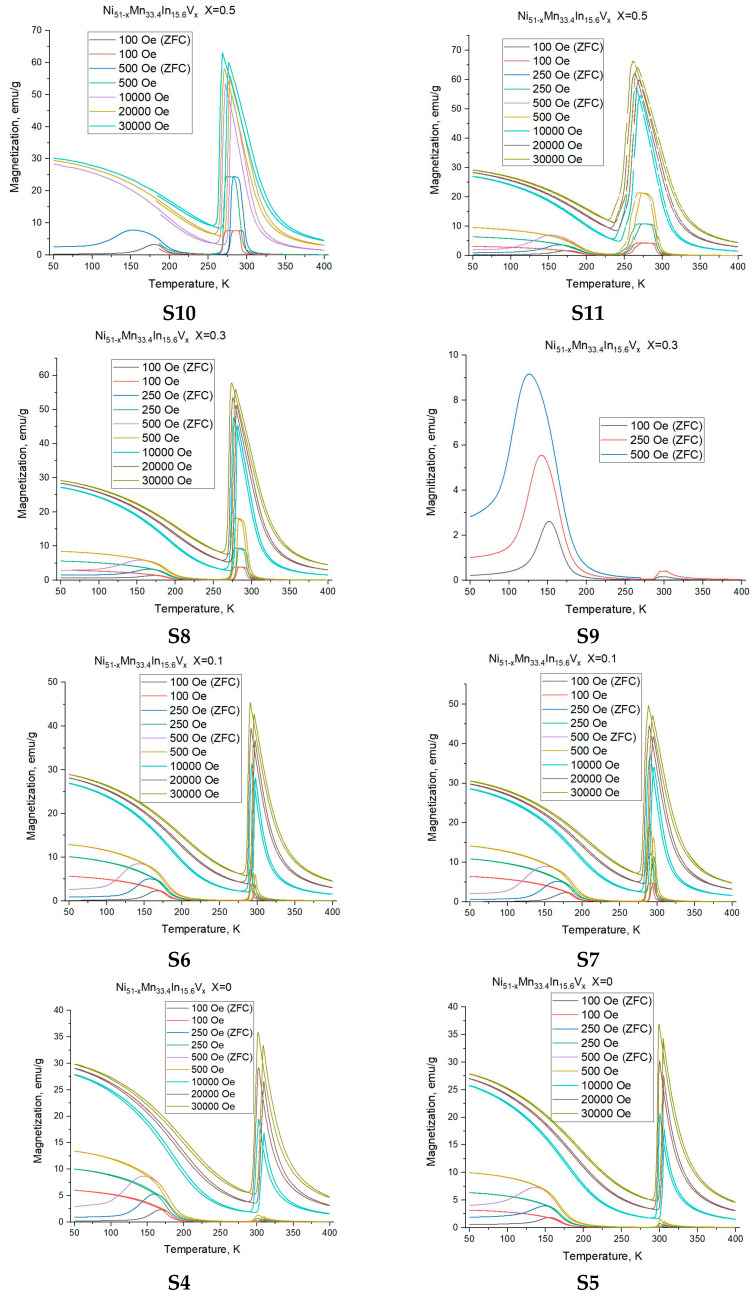
Field dependencies of the magnetization on the temperature for all the Heusler alloys studied Ni_51−x_Mn_33.4_In_15.6_V_x_ (x = 0; 0.1; 0.3; 0.5; 1).

## Data Availability

The original contributions presented in this study are included in the article. Further inquiries can be directed to the corresponding author(s).

## Appendix A

Field dependences of magnetization on temperature for all Heusler alloys Ni_51−x_Mn_33.4_In_15.6_V_x_ alloys (x = 0; 0.1; 0.3; 0.5; 1).

## References

[1] Yan H.L., Huang X.M., Esling C. (2022). Recent Progress in Crystallographic Characterization, Magnetoresponsive and Elastocaloric Effects of Ni-Mn-In-Based Heusler Alloys—A Review. Front. Mater..

[2] Bachaga T., Zhang J., Khitouni M., Sunol J.J. (2019). NiMn-based Heusler magnetic shape memory alloys: A review. Int. J. Adv. Manuf. Technol..

[3] Elphick K., Frost W., Samiepour M., Kubota T., Takanashi K., Sukegawa H., Mitani S., Hirohata A. (2021). Heusler alloys for spintronic devices: Review on recent development and future perspectives. Sci. Technol. Adv. Mater..

[4] Bekhouche A., Alleg S., Dadda K., Daoudi M.I., Saurina J., Sunol J.J. (2025). Microstructure, martensitic transformation kinetics, and magnetic properties of (Ni_50_Mn_40_In_10_)_100−x_Co_x_ melt-spun ribbons. J. Therm. Anal. Calorim..

[5] Tavares S., Yang K., Meyers M.A. (2023). Heusler alloys: Past, properties, new alloys and prospects. Prog. Mater. Sci..

[6] Krenke T., Acet M., Wassermann E.F., Moya X., Mañosa L., Planes A. (2006). Ferromagnetism in the austenitic and martensitic states of Ni−Mn−In alloys. Phys. Rev. B.

[7] Liu Z.H., Li G.T., Wu Z.G., Ma X.Q., Liu Y., Wu G.H. (2012). Tailoring martensitic transformation and martensite structure of NiMnIn alloy by Ga doping. J. Alloys Compd..

[8] Rama Rao N.V., Chandrasekaran V., Suresh K.G. (2010). Effect of Ni/Mn ratio on phase transformation and magnetic properties in Ni–Mn–In alloys. J. Appl. Phys..

[9] Chattopadhyay M.K., Sharma V.K., Chouhan A., Arora P., Roy S.B. (2011). Combined effect of hydrostatic pressure and magnetic field on the martensitic transition in the Ni_49_CuMn_34_In_16_ alloy. Phys. Rev. B.

[10] Erager K.R., Sokolovskiy V.V., Buchelnikov V.D. (2022). First-principle studies of the tendency towards segregation in Heusler alloys Ni_2_Mn_1+x_Sb_1-x_ with different atomic ordering. Phys. Solid State.

[11] Li X.-Z., Zhang W.-Y., Sellmyer D.J. (2017). Structural investigation of phase segregation in Mn_2_CrGa-based alloys. Acta Mater..

[12] Załęski K., Ekholm M., Alling B., Abrikosov I.A., Dubowik J. (2021). Local atomic configuration approach to the nonmonotonic concentration dependence of magnetic properties of Ni_2_Mn_1+x_Z_1−x_ (Z  =  In, Sn, Sb) Heusler alloys. Scr. Mater..

[13] Tian H.F., Lu J.B., Ma L., Shi H.L., Yang H.X., Wu G.H., Li J.Q. (2012). Martensitic transformation and magnetic domains in Mn_50_Ni_40_Sn_10_ studied by in-situ transmission electron microscopy. J. Appl. Phys..

[14] Das R., Sarma S., Perumal A., Srinivasan A. (2011). Effect of Co and Cu substitution on the magnetic entropy change in Ni_46_Mn_43_Sn_11_ alloy. J Appl. Phys..

[15] Zhang K., Tian X., Tan C., Guo E., Zhao W., Cai W. (2018). Designing a New Ni-Mn-Sn Ferromagnetic Shape Memory Alloy with Excellent Performance by Cu Addition. Metals.

[16] Liu J., You X., Huang B., Batashev I., Maschek M., Gong Y., Miao X., Xu F., van Dijk N., Brück E. (2019). Reversible low-field magnetocaloric effect in Ni-Mn-In-based Heusler alloys. Phys. Rev. Mater..

[17] Skripov V.P., Skripov A.V. (1979). Spinodal decomposition (phase transition involving unstable states). Usp. Fiz. Nauk..

[18] Allen S.M. (2001). Spinodal Decomposition. Encycl. Mater. Sci. Technol..

[19] Boytsova O.V., Makarevich O.N., Sharovarov D.I., Makarevich A.M. (2022). Spinodal Decomposition in the Chemistry and Technology of Inorganic Materials. Inorg. Mater..

[20] Khachaturian A.G. (1979). Theory of Phase Transitions and Structure of Solid Solutions.

[21] Kuznetsov D., Kuznetsova E., Mashirov A., Danilov D., Shandryuk G., Musabirov I., Shchetinin I., Prokunin A., von Gratowski S., Shavrov V. (2023). Influence of the Cooling Rate on Austenite Ordering and Martensite Transformation in a Non-Stoichiometric Alloy Based on Ni-Mn-In. J. Compos. Sci..

[22] Kuznetsov D.D., Mashirov A.V., Kuznetsova E.I., Prokunin A.V., Danilov D.V., Musabirov I.I., Koledov V.V., Shavrov V.G. (2024). Structural domains of austenite of non-stochiometric heusler alloys based on Ni-Mn-In. J. Radio Electron..

[23] Kuznetsov D.D., Kuznetsova E.I., Mashirov A.V., Loshachenko A.S., Danilov D.V., Mitsiuk V.I., Kuznetsov A.S., Shavrov V.G., Koledov V.V., Ari-Gur P. (2023). Magnetocaloric Effect, Structure, Spinodal Decomposition and Phase Transformations Heusler Alloy Ni-Mn-In. Nanomaterials.

[24] Kuznetsov D.D., Kuznetsova E.I., Mashirov A.V., Loshachenko A.S., Danilov D.V., Shandryuk G.A., Shavrov V.G., Koledov V.V. (2022). In Situ TEM Study of Phase Transformations in Nonstoichiometric Ni_46_Mn_41_In_13_ Heusler Alloy. Phys. Solid State.

[25] Sagaradze V.V., Belozerov Y.V., Pecherkina N.L., Mukhin M.L., Zaynutdinov Y.R. (2006). The shape memory effect in high-strength precipitation-hardening austenitic steels. Mater. Sci. Eng. A.

[26] Sagaradze V.V., Belozerov Y.V., Mukhin M.L., Zaynutdinov Y.R., Pecherkina N.L., Zavalishin V.A. (2006). A New Approach to Creation of High-Strength Austenitic Steels with a Controlled Shape-Memory Effect. Phys. Met. Metallogr..

[27] Konovalova E.V., Perevalova O.B., Koneva N.A., Smirnov A.I., Kozlov E.V. (2013). Influence of the antiphase domain structure on the long-range atomic order parameter in the Ni_3_Mn alloy with superstructure L 12. Phys. Solid State.

[28] Pushin V., Kuranova N., Marchenkova E., Pushin A. (2019). Design and development of Ti–Ni, Ni–Mn–Ga and Cu–Al–Ni-based alloys with high and low temperature shape memory effects. Materials.

[29] Sudareva S.V., Romanov E.P., Krinitsina T.P., Blinova Y.u.V., Kuznetsova E.I. (2006). Mechanism of phase transformations and fine structure of a nonstoichiometric compound YBa_2_Cu_3_O_7−δ_ at temperatures of 200 and 300 °C. Phys. Met. Metallogr..

[30] Bobylev I.B., Kuznetsova E.I., Krinitsina T.P., Zyuzeva N.A., Sudareva S.V., Romanov E.P. (2011). Restoration of the microstructure of Ba_2_YCu_3_O_7−δ_ at T > 900 °C after low-temperature decomposition. Phys. Met. Metallogr..

[31] Khachaturyan A.G., Shapiro S.M., Semenovskaya S. (1991). Adaptive phase formation in martensitic transformation. Phys. Rev. B.

[32] Kaufmann S., Niemann R., Thersleff T., Rößler U., Heczko O., Buschbeck J., Holzapfel B., Schultz L., Fähler S. (2011). Modulated martensite: Why it forms and why it deforms easily. N. J. Phys..

[33] Chulist R., Wójcik A., Sozinov A., Tokarski T., Faryna M., Schell N., Skrotzki W., Li B., Sehitoglu H., Li X. (2024). Adaptive Phase or Variant Formation at the Austenite/Twinned Martensite Interface in Modulated Ni–Mn–Ga Martensite. Adv. Funct. Mater..

[34] Zhou L., Schneider M.M., Giri A., Cho K., Sohn Y. (2017). Microstructural and crystallographic characteristics of modulated martensite, non-modulated martensite, and pre-martensitic tweed austenite in Ni-Mn-Ga alloys. Acta Mater..

[35] Lega P., Karstev A., Nedospasov I., Lv S., Lv S., Tabachkova N., Irzhak A., Orlov A., Koledov V. (2020). Blocking of martensitic transition at the nanoscale in the Ti_2_NiCu wedge. Phys. Rev. B.

